# Classifying ecosystems with metaproperties from terrestrial laser scanner data

**DOI:** 10.1111/2041-210X.12854

**Published:** 2017-08-14

**Authors:** Ian Paynter, Daniel Genest, Edward Saenz, Francesco Peri, Peter Boucher, Zhan Li, Alan Strahler, Crystal Schaaf

**Affiliations:** ^1^ School for the Environment University of Massachusetts Boston Dorchester MA USA; ^2^ Earth and Environment Boston University Boston MA USA

**Keywords:** bioinformatics, conservation, habitats, software, statistics

## Abstract

1. In this study, we introduce metaproperty analysis of terrestrial laser scanner (TLS) data, and demonstrate its application through several ecological classification problems. Metaproperty analysis considers pulse level and spatial metrics derived from the hundreds of thousands to millions of lidar pulses present in a single scan from a typical contemporary instrument. In such large aggregations, properties of the populations of lidar data reflect attributes of the underlying ecological conditions of the ecosystems.
2. In this study, we provide the Metaproperty Classification Model to employ TLS metaproperty analysis for classification problems in ecology. We applied this to a proof‐of‐concept study, which classified 88 scans from rooms and forests with 100% accuracy, to serve as a template.
3. We then applied the Metaproperty Classification Model in earnest, to separate scans from temperate and tropical forests with 97.09% accuracy (*N* = 224), and to classify scans from inland and coastal tropical rainforests with 84.07% accuracy (*N* = 270).
4. The results demonstrate the potential for metaproperty analysis to identify subtle and important ecosystem conditions, including diseases and anthropogenic disturbances. Metaproperty analysis serves as an augmentation to contemporary object reconstruction applications of TLS in ecology, and can characterize regional heterogeneity.

## 1 INTRODUCTION

**Figure 1:**
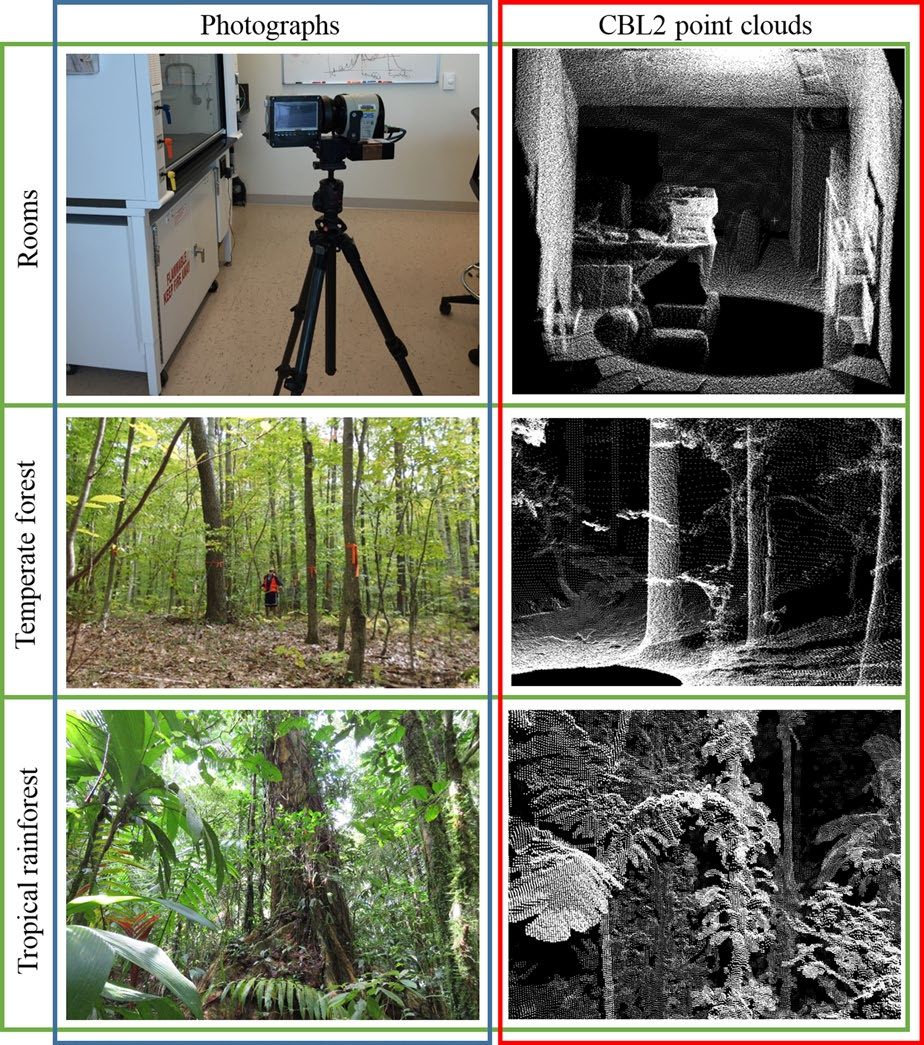
Photographs and compact biomass lidar point clouds of a Room (University of Massachusetts Boston), temperate forest (Harvard Forest) and tropical rainforest (La Selva, Costa Rica)

**Figure 2:**
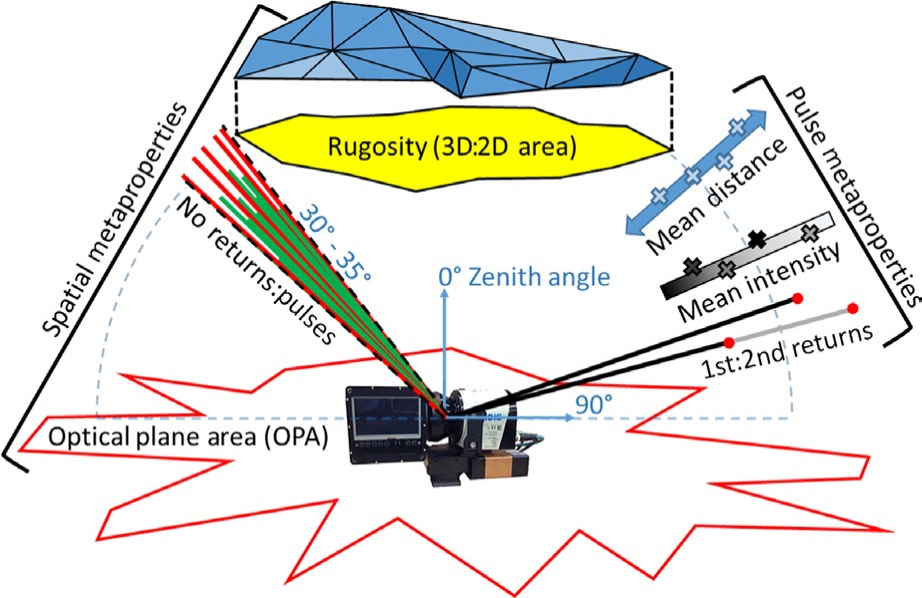
Diagram of metaproperties (descriptions in Table 4) featuring CBL2 TLS

Terrestrial laser scanner (TLS) instruments utilize Light detection and ranging (lidar) technology to emit pulses of light energy of controlled wavelengths and power. When these pulses encounter an object, back‐scattering occurs, events which are referred to as “returns”. The TLS instrument records the time‐of‐flight and intensity of the reflected signal for each return, to infer the presence, position, and reflective properties of the object (Figure 1). In this way, each lidar pulse from a TLS instrument can be thought of as a discrete sample of the three‐dimensional space around the instrument. Contemporary TLS scans often consist of hundreds‐of‐thousands to millions of lidar pulses, emitted across a range of view‐angles surrounding the instrument. Thus, properties of the large populations of pulses found in a TLS scan, such as the mean distance or intensity of the reflected signal, are direct products of the structure and positioning of objects in the surroundings of an instrument. We refer to such aggregate properties as *metaproperties* of the scan (Figure 2).

For the study of ecosystems, individual metaproperties may describe and characterize particular attributes of an ecosystem. For example, distance of returns in a forest scan could describe the spatial distribution of vegetation. By extension, groups of metaproperties describe multiple attributes of an ecosystem, and together act as a fingerprint for a scan's location. Therefore, metaproperties can classify the type of ecosystem in which a scan was taken. Furthermore, comparing metaproperties between similar ecosystems can classify ecological conditions, while metaproperties of scans within a single ecosystem can characterize spatial gradients and distinct areas.

In this study, we separate metaproperties into two types, *pulse metaproperties* and *spatial metaproperties* (Figure 2). Pulse metaproperties are population statistics of attributes of the pulses in a scan. Examples of pulse metaproperties could include the mean distance of returns in a scan or the ratio of first to second returns in pulses in a scan. Spatial metaproperties, on the other hand, are geometric attributes of the empty space between and around the objects detected by a TLS scan. This space is treated as a hypothetical object, whose geometric attributes, such as volume or cross‐sectional area, can be derived for use as spatial metaproperties. The concept of spatial metaproperties has precedent in the field of mathematical morphology. Mathematical morphology concerns the properties of objects whose shape is the empty space or medium between objects in a 3‐D space, encountered from a point in that space (Serra, 1982).

Metaproperty analysis augments contemporary TLS object reconstruction methods for studying ecosystems. Object reconstruction uses lidar data from one or more scans to construct representations of objects, such as trees, whose spatial properties, such as volume, are then measured and treated as proxies for the true objects’ ecological properties, such as biomass (Calders et al., 2015; Kaasalainen et al., 2014; Krooks et al., 2014; Raumonen et al., 2015; Romanczyk et al., 2013; Wu, Cawse‐Nicholson, & van Aardt, 2013). In this way, object reconstruction techniques refine a subset of the TLS data in one or more scans to model particular attributes of ecosystem structure. Metaproperty analysis, on the other hand, utilizes almost all of the information in each scan, to provide a holistic assessment of ecosystem structure and reflective properties.

In this paper we seek to provide several proofs‐of‐concept for the use of metaproperty analysis for ecosystem classification. We also provide a template, the Metaproperty Classification Model (MCM), for applying the methods to future studies. We evaluate the potential of metaproperty analysis for classifying ecosystems through three, increasingly subtle, binary classification problems (Figure 1). Each of these three analyses uses a group of metaproperties to predict the type of ecosystem in which TLS scans were performed. We begin by demonstrating the steps and principles of metaproperties analysis by performing the intuitively simple task of separating scans taken in rooms from those taken in forests. We then proceed to the distinguishing of tropical forests from temperate forests. Finally, we attempt to distinguish between coastal and inland tropical rainforest areas within Costa Rica.

## 2 MATERIALS AND METHODS

### 2.1 Classification problems

Three classification problems with independent TLS datasets are presented in this study (Rooms vs. Forests; Temperate vs. Tropical Forests; and Inland vs. Coastal Rainforests). Details of the sampling locations, dates, number of scans, and scanning instruments for these investigations can be found in Tables 1, 2, 3.

**Table 1:** Rooms vs. forests datasets

Dataset	Location	Year	No. of scans	Instrument
Rooms	University of Massachusetts Boston	2016	32	CBL2
Forests	Alice Holt, UK	2014	11	CBL1
	Carbono Site A1, La Selva Biological Station, Costa Rica		36	
	Delamere Forest, UK		9	

**Table 2:** Tropical vs. Temperate forests datasets

Dataset	Site	Location	Year	No. of scans	Instrument
Temperate	Hardwood	Harvard Forest, MA, USA	2014	29	CBL2
	Hemlock			13	
	Soil Moisture		2013	10	CBL1
	Snow Study			63	
Tropical	Carbono A2	La Selva Biological Station, Costa Rica	2015	231	CBL2
	Carbono A5			231	

**Table 3:** Inland vs. Coastal Rainforests datasets

Dataset	Location	Year	No. of scans	Instrument
Coastal	Sirena, Corcovado, Costa Rica	2014	431	CBL1
Inland	Carbono Sites A1, A2, A5 La Selva Biological Station, Costa Rica	2014/2015	498	CBL1/2

### 2.2 TLS data

The TLS data used in this study were acquired with the University of Massachusetts Boston Compact Biomass Lidar instruments: CBL1 and CBL2. These 905 nm, instruments acquire a scan of ~400,000 (CBL1) or ~800,000 (CBL2) pulses in 33 s, with the first and second returns recorded for each pulse. The CBL instruments have a maximum range of approximately 40 m, and a beam divergence of 15 mrad. The scanners were deployed on tripods, with the optical centre at approximately 1.3 m height.

### 2.3 Metaproperties used in this study

The metaproperties applied to the classification problems in this study are defined in Table 4 and depicted in Figure 2. There were two aims for the selection of the metaproperties for this study. The first aim was that the group of metaproperties could reasonably be expected to have explanatory power for the classification problems. We addressed this aim through the investigation of several independent preliminary datasets, not included in this study. This process particularly helped suggest which descriptive statistics might be appropriate as pulse metaproperties.

**Table 4:** Definitions of metaproperties

Name	Type	Definition	Pulse subset
Mean distance	Pulse	Mean distance to first returns	Above optical plane (≤90° zenith angle)
Mean intensity		Mean intensity of first returns	
1st:2nd returns		Ratio of first to second returns	
No return:pulses	Spatial	Ratio of the number of pulses with no returns, to total pulses emitted	Zenith angle range 30°–35°
Optical plane area (OPA)		Area of the polygon defined by joining the two‐dimensional (*X* and *Y*) Cartesian co‐ordinates of first returns from pulses emitted at the optical plane.	Optical plane (=90° zenith angle)
Rugosity		Ratio of the area of Delaunay triangulated surfaces (Lee & Schachter, 1980) fitted to the *X*,* Y* and Z co‐ordinates of returns, to the area of the polygon defined by the two‐dimensional (*X* and *Y*) Cartesian co‐ordinates of returns.	Zenith angle range 0°–30°, First returns.

The second aim was that the group of metaproperties demonstrates the diversity of metrics that can be used with metaproperty analysis, so this study can act as a pathfinder for future studies. The spatial metaproperties, which are geometric attributes of the space, primarily fulfill this aim. The selected spatial metaproperties vary in their complexity, from the simple No returns:pulses, which is computed similarly to a traditional lidar estimation of gap fraction (Strahler et al., 2008) (Table 4); to the more abstract optical plane area (OPA), which is the area of the polygon created by joining the two‐dimensional (*X* and *Y*) Cartesian co‐ordinates of all first returns from pulses emitted at the optical plane of the TLS instrument (Figure 2). In other words, the OPA is a derivation of the area of the empty space at the optical plane.

### 2.4 Metaproperty classification model

**Figure 3:**
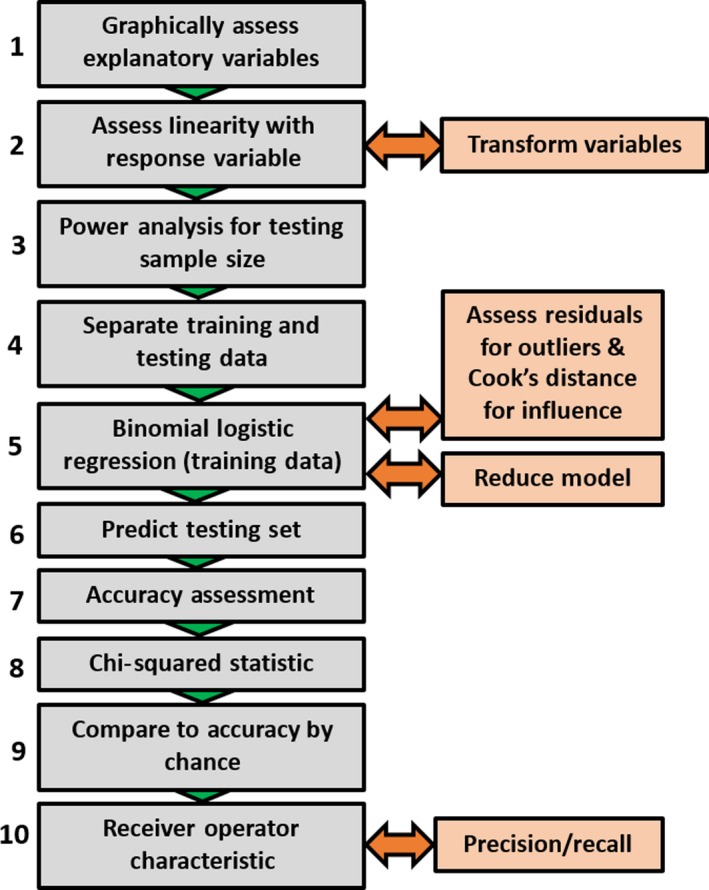
Flowchart of the steps in the Metaproperty classification model. Orange boxes denote discretionary steps to tackle specific data scenarios

We fully describe the MCM to provide a complete workflow for others wishing to apply metaproperty analysis to their own TLS lidar data and evaluate the results. The MCM is currently comprised of ten stages, with additional discretionary steps to adapt to specific data scenarios. These stages of the MCM are summarized in Figure 3 and detailed below.

The central component of the MCM for the classification problems in this study is a binomial logistic regression (stage 5), in which the selected metaproperties are the explanatory variables, with the classified locations of CBL TLS scans (Rooms vs. Forests, Temperate vs. Tropical, Inland vs. Coastal) as the binomial response variable in each case. Note that other forms of modelling, including categorical and continuous forms of regression, and other inductive classification approaches, can be substituted into the MCM as required.

#### 2.4.1 Step 1: Graphically assess explanatory variables

**Figure 4:**
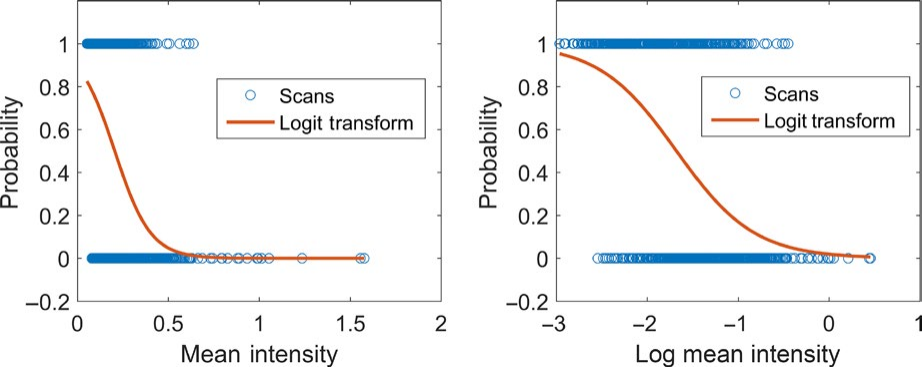
Examples of probability plots and logit transforms for the metaproperty mean Intensity in Temperate vs. Tropical Forests. Transformation can be used, as here, to improve the linearity of the variable, providing the smoothest transition of probability across the range of the variable

A scatter plot is created for each metaproperty, with the values of the metaproperty as the *X* axis and the classifications as 1 and 0 on the *Y* axis (for example, 1 = room, 0 = forest). This is overlaid with the logit transform of the classification, which is a continuous function of probabilities from 0 to 1, across the range of metaproperty values. For an example of a plot with an overlain logit transform of a variable, see Figure 4.

#### 2.4.2 Step 2: Assess linearity and separation

**Figure 5:**
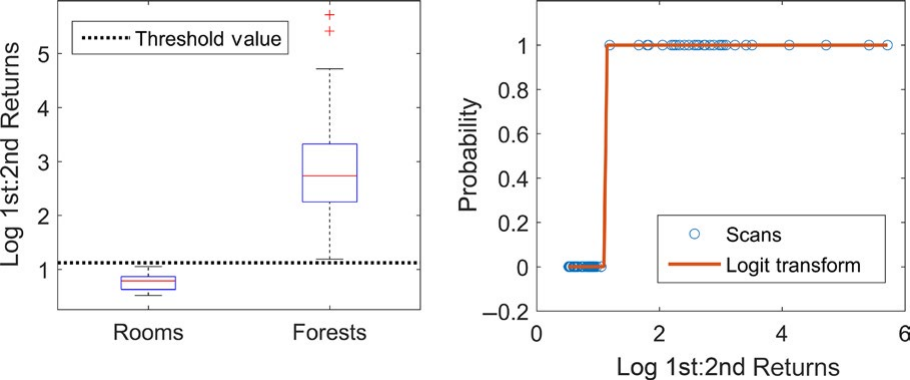
Example case where a metaproperty (1st:2nd Returns) completely separates two classification groups (Rooms vs. Forests). The probability is 0% or 100% of declaring a scan as a Forest (1). Such variables cannot be used in a binary logistic regression without employing a penalized likelihood method such as Firth's logistic regression

Each metaproperty should be assessed for the linearity of its logit transform when plotted against the response variable. It is also important to note if any metaproperty appears to completely separate the classification groups. For guidance on how to assess linearity and separation, see Figures 4 and 5. If there is no overlap between the values in the classification groups, then Firth's logistic regression, a penalized likelihood method, should be used in step 5.

#### 2.4.3 Discretionary step: Transformation

Suitable transformation (typically, natural logarithmic) can be applied if it improves linearity of the logit transformed variable.

#### 2.4.4 Step 3: Power analysis for testing sample size

A power analysis is performed to determine the required size of the *testing* dataset for 95% confidence and a 5% margin of error. This is of the form:

$$x=Z^{2}r\left(100-r\right)x$$

$$n=\frac{Nx}{\left(N-1\right)E^{2}+x},$$

where *Z* is the critical value for the confidence level (1.96 for 95% confidence), *r* is the proportion of one classification set, *N* is the total number of scans, and *E* is the margin of error (0.05).

#### 2.4.5 Step 4: Separate training and testing data

Individuals for the *testing* set should be *randomly* selected *without replacement* from the classification groups of scans, *proportional* to the groups’ representation in the total population of scans.

#### 2.4.6 Step 5: Binomial logistic regression

A binomial logistic regression should be performed on the *training* set scans, with the metaproperties as explanatory variables, and the classification for the scans as the binary response variable. If complete separation was observed in step 2 for any metaproperties, Firth's logistic regression should be used in place of standard binomial logistic regression. In either case, the Wald statistic (equivalent to a *p* value, for parameters of relational statistical models) of the β coefficient (a coefficient standardized for comparison of effect size) of the regression model should be assessed at an α value of 0.05.

#### 2.4.7 Discretionary step: Assess residuals for outliers

Individuals with high standardized residuals (>3) can be reported as outliers. If a Cook's distance test reveals that these same individuals had a disproportionate influence (Cook's distance >1.5) on the regression, then consider removing these cases and repeating the binomial logistic regression with the remainder of the *same training set*.

#### 2.4.8 Discretionary step: Reduce model

If the binomial logistic regression converges (Wald statistic of model <0.05), then the full model should be used, even if the β coefficients of one or more explanatory variables had Wald statistics exceeding the alpha value of 0.05. This guards against overfitting of the model. However, if the logistic regression fails to converge (Wald statistic of model >0.05), then consider reducing the model by iteratively removing the explanatory variable with the highest Wald statistic and rerunning the binomial logistic regression *with the same training data*, until the model converges (Wald statistic of model <0.05).

#### 2.4.9 Step 6: Predict testing set

Use the β coefficients of the model with the metaproperties of the testing set to predict probabilities and assign predicted classifications for the testing set scans.

#### 2.4.10 Step 7: Accuracy assessment

Assess the overall accuracy of the model for the training and testing sets. Calculate additional accuracy metrics which are informative to the specific classification problem. For example, true positive rate (sensitivity), true negative rate (specificity), false positive rate (recall) and false negative rate.

#### 2.4.11 Step 8: Chi‐squared statistic

Calculate the chi‐squared statistic for the results, and report the statistic along with degrees of freedom. Provided the chi‐squared statistic does not suggest a difference in the observed and expected groups (evaluated at an alpha value of 0.05), then this supports the performance of the model.

#### 2.4.12 Step 9: Compare to accuracy by chance

Calculate the accuracy by chance as the sum of the squared proportions of the number of individuals in each category to the total number of individuals in the population. This takes the form as follows:

$$\sum_{i=1}^{I}{\left(\frac{n_{i}}{N}\right)}^{2}$$

where *N* is the total number of scans, and *n*_*i*_ is the number of scans in group *i*.

#### 2.4.13 Step 10: Receiver operating characteristic (ROC)

Perform a separate ROC for the training and testing data and plot the curves together. Examine the plots, and report any localized changes in the rates of true and false positives. Report the area under curve (AUC) with 95% confidence intervals. High AUC (approaching 1) suggest the model has strong discriminatory power.

#### 2.4.14 Discretionary step: Precision and recall

If the classification groups were represented particularly unevenly, which may be reflected in anomalies in ROC, then consider performing precision and recall analysis. Visualize the precision and recall curve and report any localized changes in precision and recall rates. Report the AUC with 95% confidence intervals. Low AUC (approaching 0) suggests the model has strong discriminatory power, given the underlying distribution of individuals between the groups.

### 2.5 Transformation of explanatory variables

Graphical assessment of the explanatory variables (steps 1 and 2 of the MCM) for the Tropical vs. Temperate Forests analysis suggested that log transformation was appropriate to improve the linearity for the 1st:2nd Returns, mean intensity, OPA, and Rugosity in the Temperate vs. Tropical Forests classification problem; and for the 1st:2nd Returns, mean intensity, and OPA in the Inland vs. Coastal Rainforest classification problem.

### 2.6 Adaptations for rooms vs. forests

At step 2 of the MCM, we observed complete separation of Rooms and Forests with both the 1st:2nd Returns and no returns: pulses metaproperties. This prompted our addition of the recommendation that Firth's logistic regression be used in such cases in the future. We proceeded to form the binary logistic regression for this proof‐of‐concept classification problem without using the 1st:2nd Returns and no returns:pulses metaproperties as explanatory variables. Also, the regression for Rooms vs. Forests was trained and evaluated on the complete population of scans (no separation of training and testing sets).

## 3 RESULTS

### 3.1 Overall

The MCM formed models with greater than 80% accuracy for testing set classification prediction for all of the classification problems. The performance of the classification models declined slightly as the subtlety of the classification problems increased: Rooms vs. Forests (100% set accuracy); Temperate vs. Tropical Forests (97% testing set accuracy); and Inland vs. Coastal Rainforests (84% testing set accuracy). However, even for the most challenging classification problem, Inland vs. Coastal Rainforests, the classification derived by MCM (84%) vastly exceeded the accuracy by chance (50.22%). Additionally, two metaproperties, 1st:2nd Returns and no returns:pulses, were found to be diagnostic, each completely separating Rooms and Forests.

### 3.2 Rooms vs. Forests

The full model with four metaproperties as explanatory variables was utilized, having converged successfully (Wald statistic <0.01, 81 *df*, Table 5). There was strong evidence of a relationship between the response variable and the explanatory variables mean distance (Wald <0.01), mean intensity (Wald: 0.012), and Rugosity (Wald <0.01). There was moderate evidence of a relationship between the response variables and OPA (Wald: 0.036). There were five outliers (standardized residuals 3.2–4.1), but the distribution of standardized residuals did not suggest that these were exceptional. Cook's distance test revealed two influential individuals (Cook's distance: 4.9, 1.9), but neither were among the outliers. The model discriminated rooms from forests with 100% accuracy.

**Table 5:** Binomial logistic regression (Rooms vs. Forests)

Variable	β Coefficients	Wald statistic	*SE*
Model	−15.07	<0.01	3.92
Mean distance	0.002	<0.01	0.0006
Mean intensity	20.76	0.012	8.23
OPA	−0.0083	0.036	0.0040
Rugosity	0.12	< 0.01	0.033

### 3.3 Tropical vs. Temperate Forests

The full model was utilized, having converged successfully (Wald statistic <0.01, 398 *df*, Table 6). There was strong evidence of a relationship between the response variable and the explanatory variables Log 1st:2nd Returns (Wald <0.01) and Log OPA (Wald <0.01). There was moderate evidence of a relationship between the response variable and no returns:pulses (Wald: 0.04). The Wald statistic for mean intensity (0.05) provided little evidence of a relationship, while the Wald statistics for mean distance (0.71) and Rugosity (0.95) did not provide any evidence of a relationship with the response variable.

**Table 6:** Binomial logistic regression (Temperate vs. Tropical)

Variable	β Coefficients	Wald statistic	*SE*
Model	130.12	<0.01	23.01
Mean distance	−0.0002	0.71	0.0004
Log mean intensity	−3.07	0.054	1.59
Log 1st:2nd returns	8.64	<0.01	3.14
No return:pulses	−54.76	0.036	26.08
Log OPA	−7.72	<0.01	1.26
Log Rugosity	0.032	0.95	0.53

**Figure 6:**
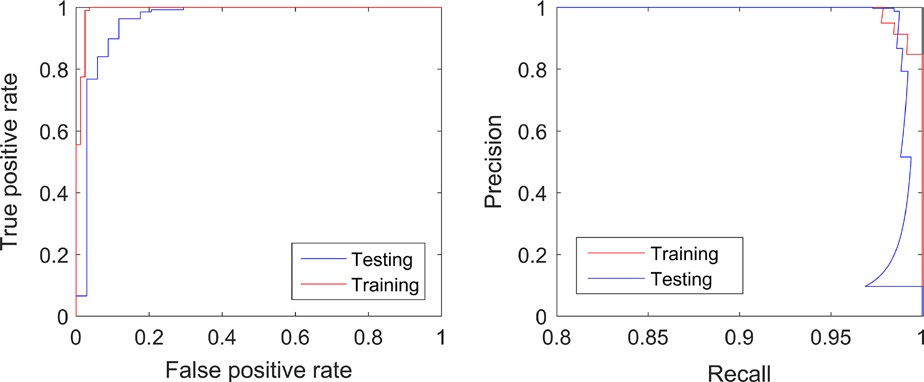
ROC curve (Left) and Precision and Recall Curve (Right) for Temperate vs. Tropical Forests classification. ROC describes changes in false positive rate as true positive rate improves. Precision & Recall describes changes in positive predictive rate (Precision) as true positive rate (Recall) improves. The wedge/step in the bottom right of the plot for the testing data indicates that a range of Recall rates were observed 0.1 Precision

There were no outliers of note (all standardized residuals <3). The model discriminated tropical from temperate forest with 98.77% accuracy in the training set, and 97.09% accuracy in the testing set, compared to 68.08% accuracy by chance. The chi‐squared statistic was 0.92 (Table 7), and the AUC of the ROC (Figure 6) was 0.989 for the training set (95% CI: 0.957–0.999) and 0.965 for the testing set (95% CI: 0.843–0.995). Due to the uneven representation of classification groups (34% temperate), precision and recall was employed (Figure 6). AUC for the training set was 0.183 (95% CI: 0.127–0.251), and AUC for the testing set was 0.196 (95% CI: 0.152–0.230).

**Table 7:** Classification model performance breakdown (Temperate vs. Tropical)

	Training set				Testing set			
	Tropical (True)	Temperate (True)	Tropical (False)	Temperate (False)	Tropical (True)	Temperate (True)	Tropical (False)	Temperate (False)
Count	322/324	78/81	3/81	2/324	138/151	70/73	3/73	13/151

### 3.4 Inland vs. Coastal Rainforests

The full model for the binary logistic regression was utilized, having converged successfully (Wald <0.01, 929 *df*, Table 8). There was strong evidence of relationships between the response variable and mean distance (Wald <0.01) and OPA (Wald <0.01). There was moderate evidence of a relationship for Rugosity (Wald: 0.04). However, the Wald statistics for mean intensity (0.13), 1st:2nd Returns (0.61) and no returns:pulses (0.55) did not provide any evidence of a relationship between these explanatory variables and the response variable. There were no outliers of note (all standardized residuals <3). The model discriminated Inland from Coastal Rainforest with 84.31% accuracy in the training set and 84.07% accuracy in the testing set, compared to 50.22% accuracy by chance. The chi‐squared statistic was 7.2301 (1 *df*, Table 9), and the AUC of the ROC was 0.9151 for the training set (95% CI: 0.892–0.941) and 0.917 for the testing set (95% CI: 0.867–0.941).

**Table 8:** Binomial logistic regression (Inland vs. Coastal)

Variable	β Coefficients	Wald statistic	*SE*
Model	−68.54	<0.01	6.99
Mean distance	0.0004	<0.01	0.0001
Mean intensity	0.77	0.16	0.50
1st:2nd returns	−0.40	0.61	0.80
No return:pulses	−7.26	0.55	12.24
OPA	3.70	<0.01	0.35
Rugosity	−0.0097	0.044	0.0048

**Table 9:** Classification model performance breakdown (Inland vs. Coastal)

	Training set				Testing set			
	Inland (True)	Coastal (True)	Inland (False)	Coastal (False)	Inland (True)	Coastal (True)	Inland (False)	Coastal (False)
Count	298/346	250/304	54/304	48/346	116/144	111/126	15/126	28/144

## 4 DISCUSSION

### 4.1 Performance of metaproperty analysis and the MCM

The classification models formed by metaproperty analysis, following the structure of the MCM, had strong predictive power. The classification models demonstrated 100% accuracy for separating Rooms vs. Forests, 97% testing set accuracy for delineating Temperate vs. Tropical Forests, and 84% testing set accuracy when predicting Inland vs. Coastal Rainforests. The observed accuracy of each of these models exceeded their expected accuracy by chance. In each classification problem, a different subset of metaproperties was found to have explanatory power. Furthermore, each metaproperty had explanatory power in at least one of the classification problems (Table 10).

**Table 10:**
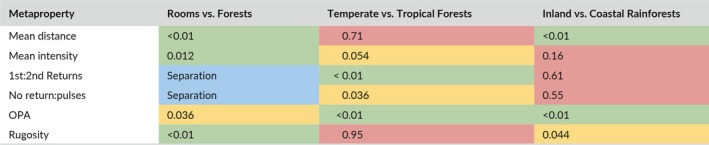
Wald statistics of explanatory metaproperties in classification problems. Green indicates strong evidence of relationship between a metaproperty and the response variable of the classification (Wald statistic <0.02). Yellow indicates moderate to weak evidence (Wald statistic: 0.02–0.06). Red indicates no evidence (Wald statistic >0.06). Blue indicates a metaproperty completely separated classification groups (100% accuracy)

For the Rooms vs. Forests classification, we expected strong discrimination of Rooms from Forests. The Rooms vs. Forests classification problem was conducted primarily as a demonstration of metaproperty analysis, as well as a test for the workflow of the MCM. Regardless, unilateral separation (100% accuracy) was possible based on either of two individual metaproperties (1st:2nd Returns and no returns:pulses), as well as the combined explanatory power of the rest of the metaproperties. It is important to remember that since the regression for Rooms vs. Forests was both trained and evaluated on the complete population of scans, confidence in this performance should be reduced accordingly.

**Figure 7:**
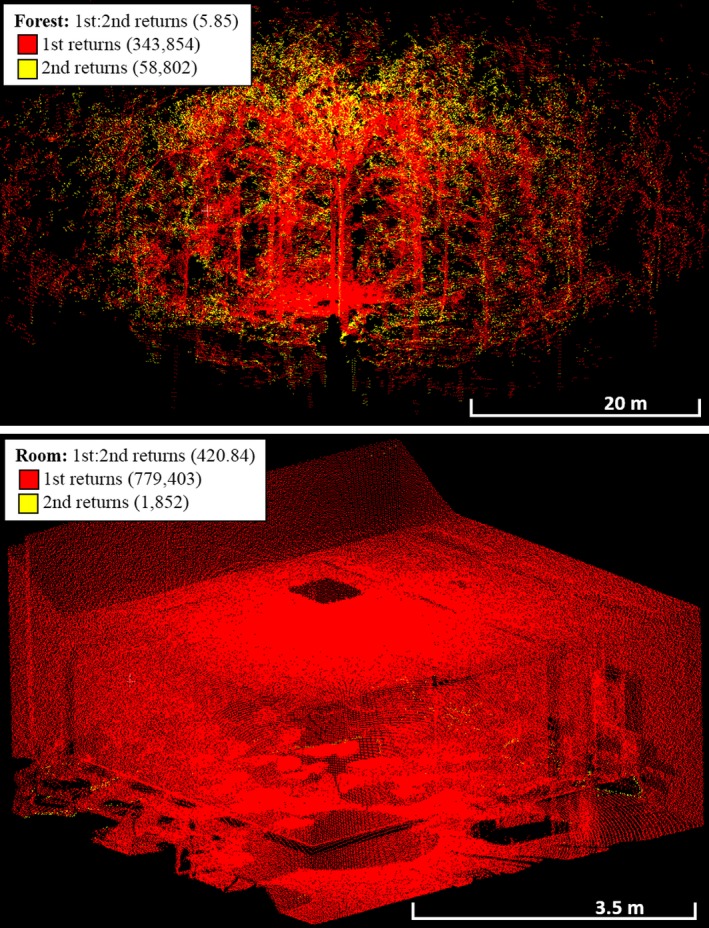
CBL point clouds showing the metaproperty 1st Returns and 2nd Returns, which was consistently higher in Rooms than in Forests

**Figure 8:**
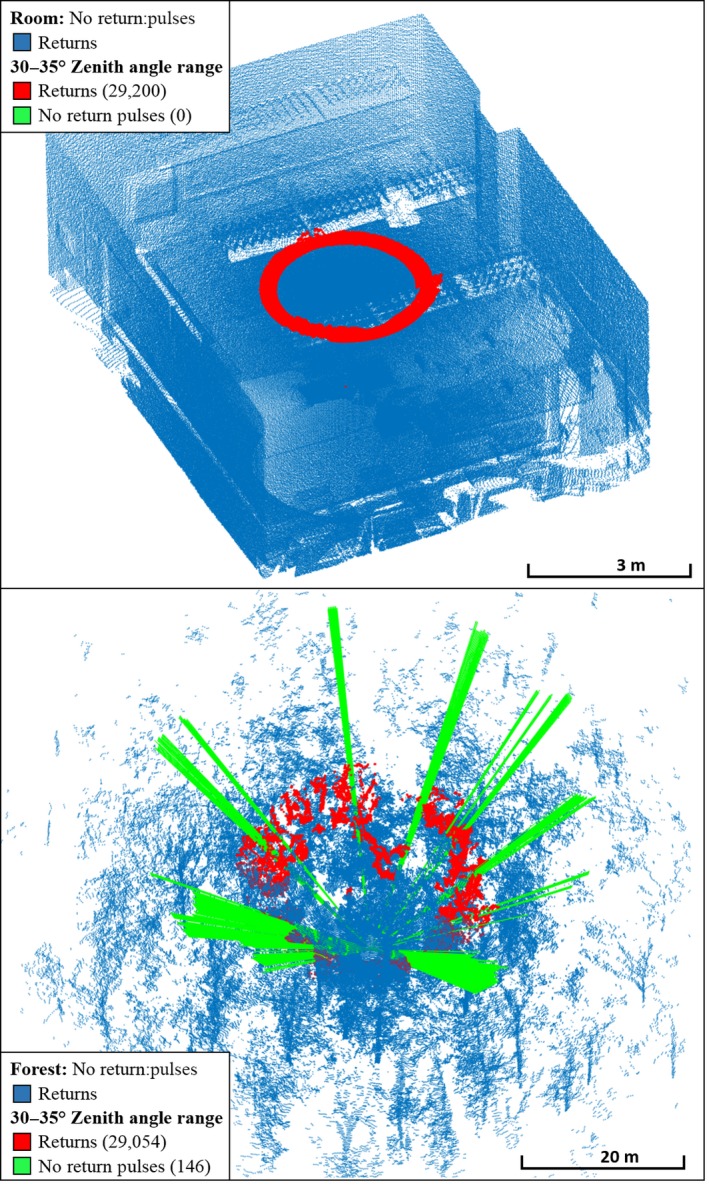
CBL point clouds for no returns in Rooms vs. Forests. Forests had consistently higher no returns:pulses than Rooms

The metaproperty 1st:2nd Returns was a diagnostic property for separating Rooms from Forests, as it was higher in Forests than in Rooms 100% of the time (Figure 7). This likely resulted from the solid surfaces of the Rooms providing only first returns, except where object edges partially intercepted pulses, while the relatively fragmented structure of the Forests, consisting of many small components such as leaves and twigs (Figure 7), provided far more second returns. The metaproperty of no returns:pulses also completely separated Rooms from Forests, with more pulses without returns consistently observed in Forests. An intuitive explanation is that Rooms have continuous ceilings, while Forest canopies have gaps in their complex structure (Figure 8). Mean Intensity had a large effect size (β: 20.76) and was a strong discriminator (Wald: 0.01). This is likely due to Rooms being enclosed spaces, comprising mostly continuous, reflective, hard surfaces that reflect the entire footprint of each pulse. Forests, on the other hand, comprise objects which are generally smaller, less reflective, and have more surface variation, lowering the amount of energy reflected from each pulse.

**Figure 9:**
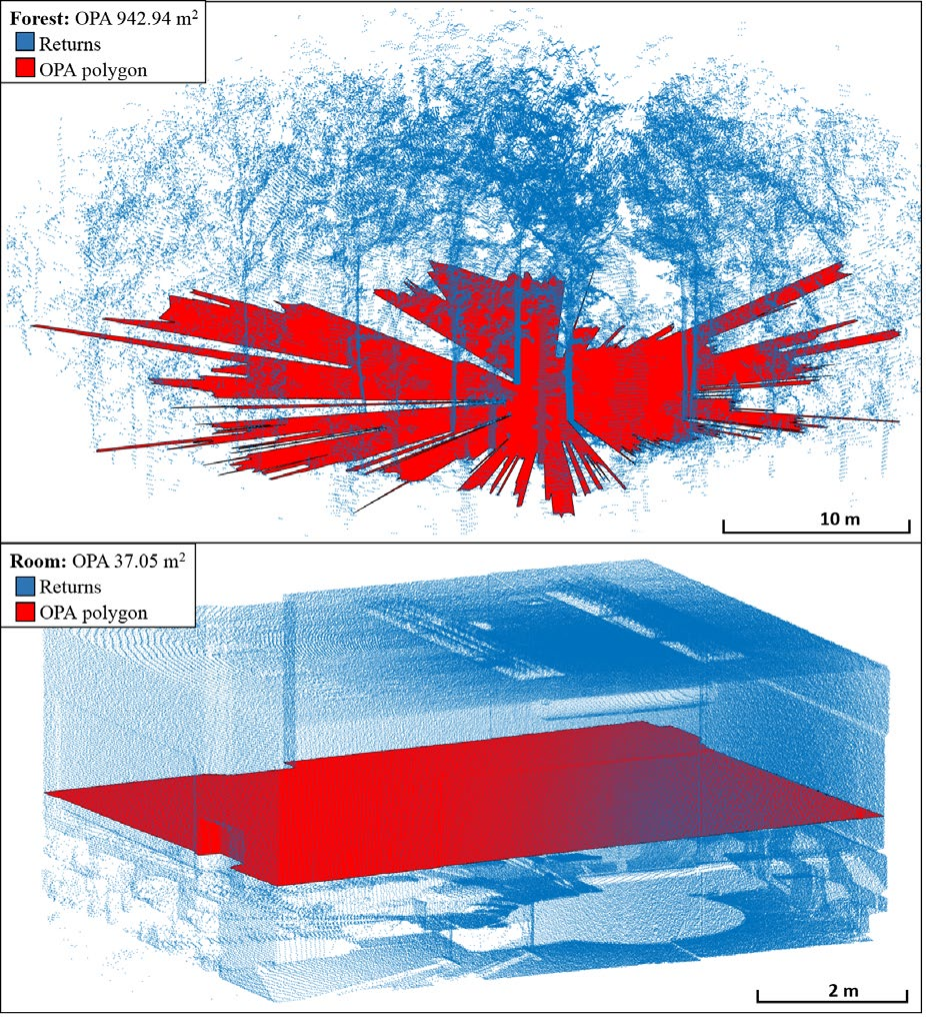
OPA for Rooms vs. Forests. Forests consistently had higher OPA than Forests. OPA, optical plane area

Rooms demonstrated a tendency towards higher mean distance and lower OPA (Figure 9) than Forests. Even though these had very small β coefficients, suggesting a low magnitude of effect, the existence of the relationships was strongly supported by the Wald statistics (Table 5). The lower mean distance in Forests could be explained by the abundance of near‐field vegetation and tree trunks, with the increased OPA resulting from the long sight‐lines between these objects (Figure 9). The many long sight‐lines of Forests could have been expected to increase mean distance, but sight‐lines that exceeded the maximum range of the CBL, or corresponded to gaps in the canopy, did not contribute to mean distance, since the metaproperty only considers pulses with first returns. Rugosity was higher (β: 0.117) in Rooms than Forests. This counterintuitive result is discussed below, but Rugosity still provided extremely strong discriminatory power (Wald <0.001), and thus was still valuable for the task of classifying Rooms from Forests.

Classifying Tropical Forests from Temperate Forests is also an easy task, with many potential diagnostic properties, including such basic information as the latitude of a scan's location. Given that metaproperties draw distinctions between ecosystems based on the integration of a large amount of spatial and reflective information captured by lidar data, the 97% testing set accuracy is encouraging. However, the rare misclassified cases (16/224, Table 7) may warrant investigation.

**Figure 10:**
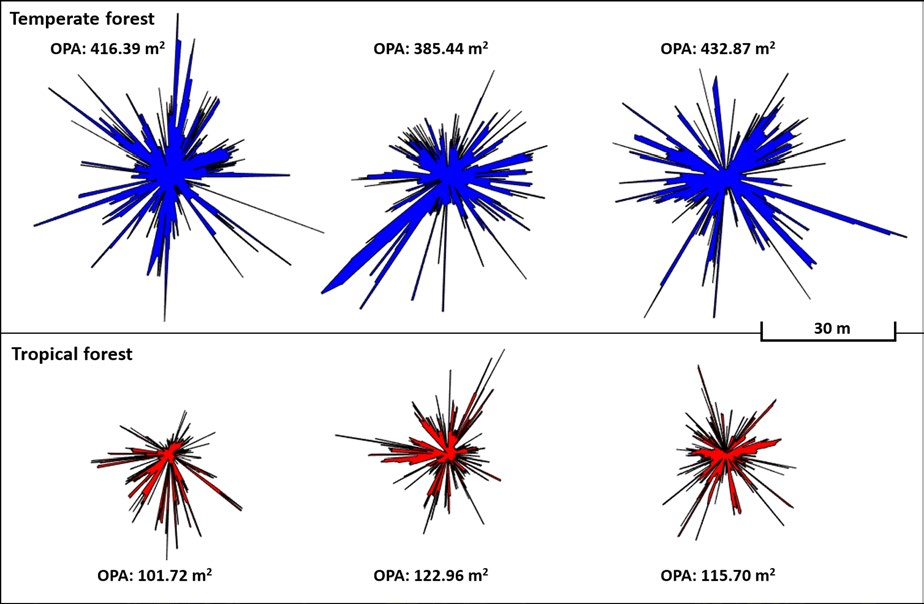
OPA polygons for Temperate vs. Tropical Forests. Temperate Forests tended towards higher OPA

There was strong evidence for the ability of Log 1st:2nd Returns and Log OPA to discriminate between Temperate Forests and Tropical Forests (Table 6). The higher frequency of second returns in Tropical forests (β: 8.64) could be attributed to the dense, leafy understory and generally denser vegetation, resulting in more partial interceptions of pulses in the near‐field, and providing many additional targets for the remaining portions of pulses. The lower OPA in Tropical Forests (β: −7.72) could be attributed to the same vegetation features, since the more frequent and larger gaps between objects in Temperate Forests greatly increase the area of the optical plane (Figure 10).

**Figure 11:**
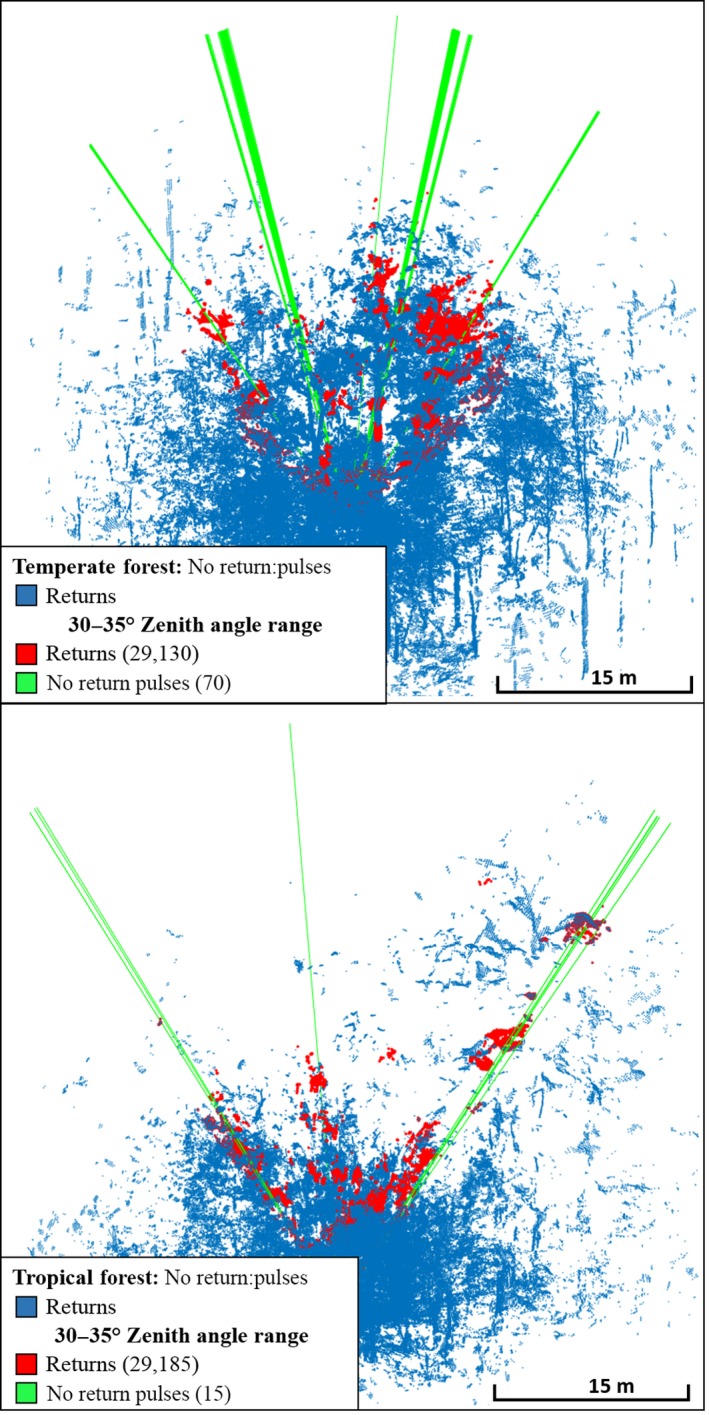
CBL point clouds showing no returns:pulses, which tended to be higher in Temperate Forests than in Tropical Forests, attributable to the denser sub‐canopy layer of Tropical Forests, which intercepts many pulses

There was also reasonable evidence for discrimination based on no returns:pulses (Wald: 0.035), which indicated a strongly negative relationship with Tropical Forests (β: −54.76). The subcanopy of Tropical Forests likely accounts for this (Figure 11), as it consists of broad‐leafed plants that intercept more pulses within the range of the instrument. There was a weak indication (Wald: 0.054) that the Mean Intensity may have been lower in the Tropical Forest group. This may have resulted from a relatively higher moisture content, leading to the absorption of pulse energy at the 905 nm wavelength of the instrument.

The Coastal vs. Inland Rainforest classification was a more rigorous test of the capabilities of metaproperty analysis. While there are still direct ways to diagnose these ecosystems, including by geographical location, they are structurally much more similar than Temperate and Tropical Forests. The MCM did not perform as well as for the other classification problems, but the testing accuracy was still 84.07%. It should be noted that the high chi‐squared statistic (7.23, *p* < .01) indicates that the observed level of error in the model was not a function of chance. Therefore, improvements to this classification model should only be expected with metaproperties with more explanatory power, or more representative training data. Regardless, this classification problem shows metaproperty analysis and the MCM successfully performing the function for which they were created: separating an ecosystem condition that is reflected only in the integration of small differences in ecosystems attributes.

**Figure 12:**
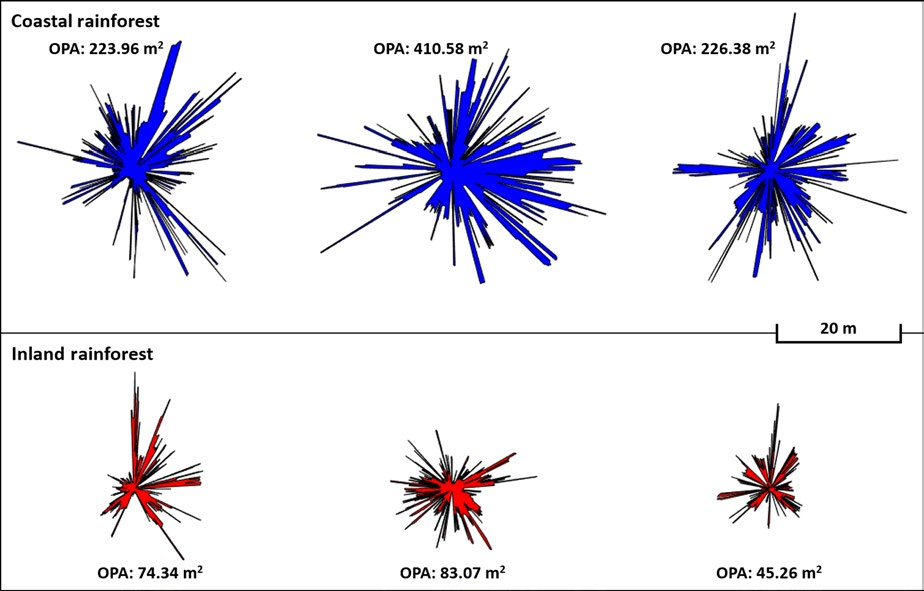
OPA polygons for Inland vs. Costal rainforests, plotted to scale. Coastal Rainforests tended towards higher OPA

Mean Distance, OPA, and Rugosity (noting the borderline Wald statistic of 0.04) were found to have explanatory power for separating Inland Rainforests from Coastal Rainforests. OPA was much higher in Coastal Rainforest (β: 3.7) than Inland Rainforest. The lower OPA of Inland Rainforests suggests the presence of a more dense subcanopy, further supported by the slightly but consistently higher Mean Distance (β < 0.01, Wald <0.01). The OPA profiles for Coastal Rainforest and Inland Rainforest were similar in form, but with far smaller overall extent in the Inland group (Figure 12). The Inland Rainforest may have more vertical variation due to both the prevalent subcanopy, and the relative abundance of epiphytic plants on the trunks of trees (Merwin, Rentmeester, & Nadkarni, 2003). This vertical variation may account for its increased Rugosity (β: −0.0097), since height variation in lidar returns increases the 3D area.

### 4.2 Selection of metaproperties

There are many possible metaproperties that could be extracted from TLS scans, and these could be used in many different combinations. Selecting an appropriate metaproperty, or set of metaproperties, is therefore a challenging process. Sometimes a particular metaproperty will be hypothesized a priori to explain a particular, measurable ecosystem condition. For example, one might hypothesize that the number of laser pulses with multiple returns might be large in conifer forests, given their fine needles. In such cases, the single metaproperty can simply be extracted and the relationship to the ecosystem condition can be tested via traditional inferential statistical techniques such as linear regression.

However, when metaproperties are being used for more exploratory studies where no particular relationships are hypothesized a priori, as in this paper, a suite of metaproperties is desirable. The set of metaproperties to be included in an exploratory study should ideally be determined in an independent, but ecologically similar, preliminary dataset. Examining multiple potential combinations in the main dataset to select metaproperties is to be avoided, as this sort of “data snooping” drastically decreases confidence in any relationships that are eventually observed.

In the absence of a preliminary dataset, we can still guide the a priori selection of metaproperties with several general considerations. Firstly, a group of metaproperties should include pulse metaproperties that utilize as much of the information captured in the lidar pulses of the relevant TLS instrument as possible. TLS instruments other than the CBL may capture more returns per pulse or full waveform data (Calders et al., 2015), or return intensity at multiple wavelengths (Douglas et al., 2012; Gaulton, Danson, Pearson, Lewis, & Disney, 2010; Howe et al., 2015), resulting in many potential pulse metaproperties. In general, pulse metaproperties will take the form of descriptive statistics of the entire population of pulses, such as the mean, minimum, maximum, standard deviation, range, or ratio. In this study, the particular statistics used as pulse metaproperties were partly chosen to provide some resilience against “ecosystem scaling.” Ecosystem scaling occurs when objects are different in physical size, but not general morphology, such as dwarf vs. tall forests.

Secondly, spatial metaproperties should be independent of each other, since explanatory variables in a binomial logistic regression are assumed to be independent. Independence, in this case, means that the spatial metaproperties should not consider the same geometric properties or regions of the empty space. Additionally, spatial metaproperties should also not be substantially dependent on pulse metaproperties, such that they obviously co‐vary. Of course, an understanding of the technology makes it clear that all lidar metaproperties are, at some level, interdependent. Thus, the aim is to employ metaproperties that are influenced far more by the attributes of the ecosystem than by each other. The spatial metaproperties chosen for this study, for example, consider the peripheral geometric properties of the empty space, and therefore interact minimally with the pulse metaproperties.

Metaproperties may also have dependencies on the attributes of particular TLS instruments. For example, the CBL has a wider beam divergence than other contemporary TLS (15 mrad). This attribute may have a notable influence on the metaproperty of no returns:pulses, since wider beams are more likely to encounter an object, reducing the frequency of gap observation. Another example of a scanner attribute influencing a metaproperty was apparent in the Rugosity values of Rooms vs. Forests. The flat ceilings of Rooms would be intuitively expected to have less complex 3D structure, and therefore lower Rugosity values, than Forests. However, Rugosity values were generally higher (β: 0.117) in Rooms than in Forests.

This counterintuitive result stems from the variation in the range estimation of returns in CBL pulses (±50 mm). This instrument‐specific range variation creates a large amount of 3D structure out of a relatively flat surface such as a Room ceiling. Conversely, in Forests, the CBL range variation is mitigated by the natural variation in the vertical structure of the canopy. In addition, the pulses that form Rugosity are emitted at a range of zenith angles (0°–30°), and therefore have a larger spread, and thus a larger 2D area, with distance. Since the ceilings of Rooms are much lower than Forest canopies, the 2D areas for calculating Rugosity were often much smaller in Rooms, further increasing the Rugosity values.

This discussion of interactions between scanner and ecosystem attributes highlights the dependence of metaproperties on both the structural properties of the ecosystem being studied and the idiosyncrasies of the instrument used for the study. Therefore, results should always be contextualized by the attributes of the instrument, as in this study. It should be noted that scanner idiosyncrasies are a systematic source of variation, and therefore they are not detrimental to analyses that use a single instrument. However, in the case of analysing combined datasets from multiple instruments, caution will be necessary.

### 4.3 Independence of TLS data in overlapping scans

Within a logistic regression, individual samples of explanatory variables, which in this study are individual TLS scans, are assumed to be independent. This raises the question of whether TLS scans can be considered independent if they are acquired close together, and therefore interact with common regions of space, since characteristics of common regions are then included in the metaproperties of multiple scans, violating their independence. There is spatial overlap of this nature for many of the scans in this study. However, due to the discontinuous sampling and line‐of‐sight limitation of TLS instruments, the values of metaproperties for a location are heavily dependent on the specific position of the TLS instrument. This considerably mitigates concerns about scan independence. The dependence of metaproperties on viewing angle could be investigated empirically, but the conservative option for future studies would be to avoid spatial overlap of scans.

## 5 CONCLUSIONS

In this study, we introduced metaproperties as metrics that aggregate the spatial and reflective information in lidar data. We established metaproperty analysis as a way to effectively utilize the increasing variety and quality of information from contemporary TLS instruments to classify ecosystems. Through a series of ecosystem classification problems, we demonstrated how metaproperty analysis can find individual, powerful indicators for ecosystem type, as well as weighing more subtle evidence from multiple metaproperties. The MCM provided a complete workflow for ecosystem investigation, including considerations of statistical power, optimization of models, presence and influence of outliers, and appropriate metrics to assess model accuracy. The discretionary steps of the MCM are adaptable to a range of data scenarios.

Since metaproperty analysis can simultaneously consider many attributes of an ecosystem, it can uncover single diagnostic properties or form predictive models based on multiple metaproperties for ecosystem types and conditions. This could improve characterization of conditions which are currently challenging for ecological assessment, such as disease, storm damage, and anthropogenic disturbances. Metaproperty analysis may be particularly useful for studying diseases and infestations in their early stages. While these conditions eventually result in easily identifiable changes in ecosystems, effective management relies on their classification in early stages, when the changes are more subtle. Metaproperty analysis could help establish patterns of spatial heterogeneity within ecosystem, which could guide appropriate stratified sampling for validation of airborne and satellite observations. Metaproperty analysis methods can also be applied to historical lidar data, providing a baseline for observing ecosystem change.

The emerging class of TLS instruments that are optimized for rapid scanning and portability, such as the Compact Biomass Lidar (CBL), synergize well with metaproperty analysis. Favourable deployment logistics enable the capture of many TLS scans across large areas of ecosystems (Paynter et al., 2016). The resulting increase in sample size compared with previous instruments improves the inferential power of metaproperty analyses. A large number of scans can also provide subsets of data for preliminary analyses, yielding refined groups of metaproperties or candidates for diagnostic metaproperties for ecosystem conditions. Consideration of preliminary studies could be added to the MCM as a discretionary step. However, targeting reduced groups of metaproperties also warrants caution, as overfitting analyses to a current set of observations may exclude metaproperties with explanatory power for future observations and conditions.

Metaproperty analysis also reduces lidar data to a lightweight format, which improves the accessibility of the techniques, and thus encourages large‐scale and collaborative ecosystem studies. To encourage collaboration, and maximize use of historical data, we must facilitate the combination of datasets from different TLS scanners. Adapting metaproperty analysis for use with airborne lidar data could also be extremely useful to achieve ecosystem assessment over larger spatial extents. The independence of metaproperties, and the independence of overlapping scans, also remains important topics for further investigation. However, metaproperty analysis techniques have the potential to be a pathfinder for transitioning TLS sampling from the plot scale to the landscape scale.

## AUTHORS’ CONTRIBUTION

I.P., D.G., E.S., F.P. and Z.L. collected TLS data. I.P. and D.G. conducted data analysis. I.P., D.G., E.S. and C.S. designed the study and produced the manuscript. Z.L. and A.S. offered additional refinements to the design of the study and manuscript. P.B. provided refinements to the study presentation and manuscript during the resubmission process.

## DATA ACCESSIBILITY

The Compact Biomass Lidar (CBL1 and CBL2) scans utilized in this study are available via FTP at: ftp://rsftp.eeos.umb.edu/data02/OpenAccessPaperData/cbl-metaproperty/Metaproperties_Data.zip
